# Quantitative expression data of human estrogen receptor α variants in non-functioning pituitary adenomas obtained by reverse transcription-digital polymerase chain reaction analysis

**DOI:** 10.1016/j.dib.2020.106452

**Published:** 2020-10-22

**Authors:** Yujiro Hattori, Hirotaka Ishii, Shigeyuki Tahara, Akio Morita, Hitoshi Ozawa

**Affiliations:** a Department of Anatomy and Neurobiology, Graduate School of Medicine, Nippon Medical School, Tokyo, Japan; b Department of Neurological Surgery, Nippon Medical School, Tokyo, Japan

**Keywords:** non-functioning pituitary adenomas, *ESR1*, estrogen receptor α variant, RT-dPCR

## Abstract

Expression profiles of gonadal steroid receptor variants have been reportedly associated with malignancy in breast and prostate cancers [1,2]. However, such associations with pituitary tumors remain unclear. Therefore, the expression levels of the wild-type *ESR1* (ERα66) and the *ESR1* variants (ERαi34, ERαi45c, and ERαΔ5) transcripts encoding constitutively active ERα proteins with C-terminal truncation in non-functioning pituitary adenomas (NFPAs) were evaluated using reverse transcription-digital polymerase chain reaction. The results revealed that the expression levels of the variants were approximately two orders of magnitude lower than that of ERα66 in NFPAs. These data were based on our previous article entitled “Accurate assessment of estrogen receptor profiles in non-functioning pituitary adenomas using RT-digital PCR and immunohistochemistry” [3].

## Specifications Table

**Table:** 

Subject	Endocrinology
Specific subject area	Estrogen receptor α variant
Type of data	Figure, Table
How data were acquired	QuantStudio 3D Digital PCR System platform with a GeneAmp® PCR System 9700 (Thermo Fisher Scientific, Massachusetts, USA)
Data format	Schematic, Analyzed, Raw
Parameters for data collection	RT-dPCR data were acquired according to the manufactures’ protocols.
Description of data collection	Total RNA was extracted from non-functioning pituitary adenomas to perform RT-dPCR.
Data source location	Institution: Graduate School of Medicine, Nippon Medical School City/Town/Region: Bunkyo-ku, Tokyo Country: Japan Latitude and longitude (and GPS coordinates, if possible) for collected samples/data: 35.719523, 139.761790
Data accessibility	With the article
Related research article	Y. Hattori, H. Ishii, S. Tahara, A. Morita, and H. Ozawa, Accurate assessment of estrogen receptor profiles in non-functioning pituitary adenomas using RT-digital PCR and immunohistochemistry, *Life Sciences*, 260 (2020) 118416. (https://doi.org/10.1016/j.lfs.2020.118416)

## Value of the Data

• Expression profiles of gonadal steroid receptor variants have been reportedly associated with malignancy in breast and prostate cancers. In these tumors, the expression levels of the variants that are not normally detected were increased as compared to the wild-type.
• To date, several studies have reported that the wild-type full-length estrogen receptor (ER) is involved in the development of non-functioning pituitary adenomas, but there have been no such reports for ER variants.
• The data obtained in this study will be valuable to researchers with interests in endocrinology and steroid hormone receptors, especially ERs.
• In this study, a quantitative method was established to evaluate the expression levels of human *ESR1*. Furthermore, this method can be used not only for pituitary tumors but also for tumors originating in other organs.

## 1 Data Description

**Fig. 1:**
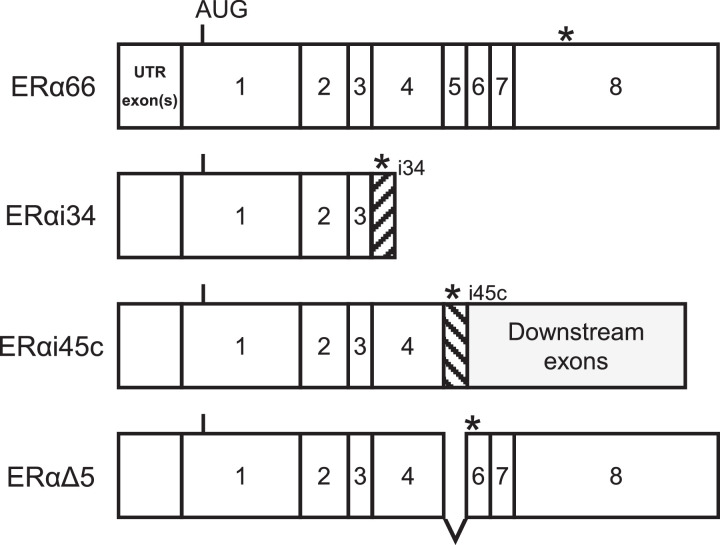
Schematic structures of *ESR1* variant mRNAs.

The human *ESR1* gene contains eight conventional coding exons (exons 1–8) and several cryptic exons. Alternative splicing of the exons generates multiple *ESR1* variants with distinct structures and functions [4,5]. ERαi34 [5], ERαi45c [4,6], and ERαΔ5 [4,7] variant transcripts encode C-terminally truncated ERα proteins with strong constitutive activation. The mRNA structures of wild-type *ESR1* (ERα66) and the *ESR1* variants are represented schematically in Fig. 1. To the best of our knowledge, this is the first report examining the expression levels of *ESR1* variants in non-functioning pituitary adenomas (NFPAs). In the present study, the expression levels of the variant transcripts and wild-type *ESR1* were compared.

The mRNA structures of wild-type *ESR1* (ERα66) and *ESR1* variants (ERαi34, ERαi45c, and ERαΔ5) are represented schematically. The AUG codon sequences and asterisks indicate the translational initiation and termination sites, respectively. The hatched boxes indicate cryptic exons/sequences.

**Fig. 2:**
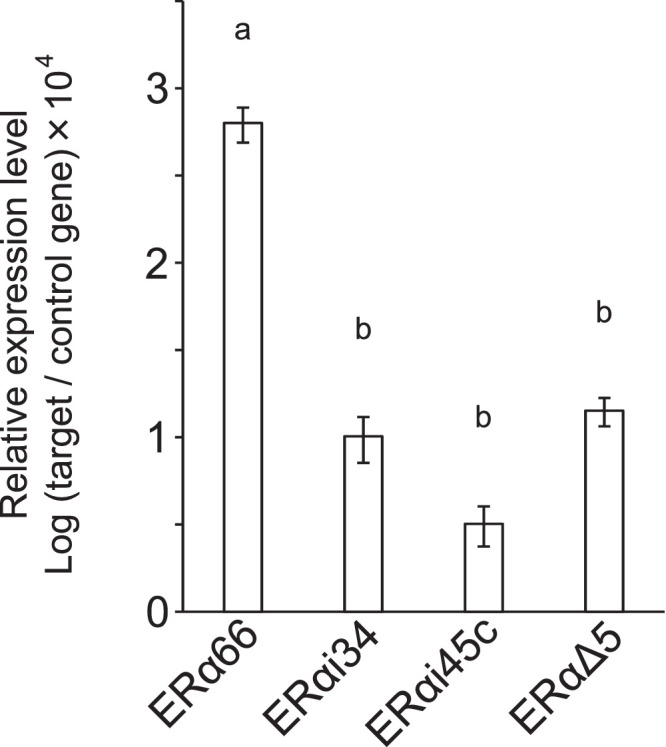
mRNA expression levels of *ESR1* variants in NFPA tissues.

The expression levels of the ERα66 and variant transcripts in NFPAs were quantified using reverse transcription-digital polymerase chain reaction (RT-dPCR) (Fig. 2, Table 1). The expression values were determined by dividing the copy number of the target gene by the geometric mean of the copy numbers of the internal control genes, *GAPDH* and *ALAS1*. The normalized values (mean ± SEM) of ERα66, ERαi34, ERαi45c, and ERαΔ5 were 0.063 ± 0.014, 0.001 ± 0.0002, 0.0003 ± 0.00008, and 0.001 ± 0.0002, respectively. In NFPA tissues, the expression levels of the variants were approximately two orders of magnitude lower than that of ERα66.

mRNA expression levels of wild-type *ESR1* (ERα66) and the *ESR1* variants (ERαi34, ERαi45c, and ERαΔ5) in NFPA tissues were quantified by RT-dPCR analysis. Data are expressed as mean ± SEM. The expression levels were normalized against the combination of the internal control genes, *GAPDH* and *ALAS1*. The columns with different letters indicate significant differences. Raw data are shown in Table 1.

**Table 1m:** Raw gene expression data obtained by RT-dPCR analysism

	mRNA copies/μL					
Case No.	ERα66	ERαi34	ERαi45c	ERαΔ5	ALAS1	GAPDH
1	1123.1	0.74	0.713	25.737	288.19	363.82
2	157.57	1.147	0.566	1.923	37.752	82.3
3	1785.7	0.168	1.684	19.601	488.26	828.22
4	391.06	1.836	0.45	4.962	83.877	272.06
5	1038.9	0.175	2.717	6.612	301.7	632.52
6	941.3	4.968	0.69	15.204	307.13	651.61
7	277.26	10.416	1.392	4.602	83.579	233.62
8	80.122	0.645	0.527	2.068	32.551	51.41
9	926.98	0.455	0.617	10.196	303.21	782.61
10	267.47	0.539	0.496	4.23	254.06	608.79
11	43.512	7.284	0.438	6.126	70.703	207.68
12	189.19	1.278	1.076	4.472	460.83	987.49
13	177.62	1.194	0.882	3.75	435.84	1247.1
14	119.74	0.349	0.122	0.699	424.47	1073.2
15	26.577	10.86	5.466	1.668	166.41	414.39
16	6.853	1.128	0.432	7.998	99.642	329.89
17	2.203	2.087	0.631	4.625	56.567	97.117
18	1.978	6.946	2.308	0.829	54.595	143.65
19	1.968	3.9	0.684	1.344	87.436	305.66
20	3.048	0.58	0.437	0.502	248.43	530.42

## 2 Experimental Design, Materials and Methods

### 2.1 Sample preparation

NFPA specimens were collected from 20 patients with a definitive pathological diagnosis. Detailed patient information is reported in our related research article [3]. Total RNA was extracted from the NFPAs immediately after resection using NucleoSpin® RNA Plus kits (Macherey-Nagel GmbH & Co. KG, Düren, Germany) and reverse-transcribed using ReverTra Ace® reverse transcriptase (Toyobo Co., Ltd., Osaka, Japan). The reverse transcription reaction was conducted at 42°C for 60 min and then terminated by heating at 75°C for 15 min.

### 2.2 RT-dPCR analysis

For expression analyses, dPCR was performed with the QuantStudio 3D Digital PCR System platform and a GeneAmp® PCR System 9700 (Thermo Fisher Scientific, Waltham, MA, USA). The dPCR primers were synthesized by Thermo Fisher Scientific. The TaqMan Gene Expression Assay identification codes of the primers were AI1RXEC for *ESR1*, AI39TQS for ERαi34, AI20VKK for ERαi45c, AI0IY74 for ERαΔ5, Hs99999905_m1 for *GAPDH*, and Hs00963537_m1 for *ALAS1*. Each dPCR reaction comprised cDNA corresponding to 150 ng of total RNA and 0.75 μl of TaqMan Genotyping Master Mix in accordance with the manufacturer's protocol. The PCR mixture (14.5 μl) was loaded onto each QuantStudio 3D digital PCR chip (Thermo Fisher Scientific). The cycling condition of the dPCR reaction comprised an initial denaturing step at 96 °C for 10 min, followed by 39 cycles at 60 °C for 2 min and 98 °C for 30 s, and a final extension step at 60 °C for 2 min, as described previously [3].

### 2.3 Statistical Analysis

All statistical analyses were performed using IBM SPSS Statistics for Windows, version 25.0 (IBM Corporation, Armonk, NY, USA). The data were assessed using one-way analysis of variance followed by Tukey's post-hoc test. A *P*-value of less than < 0.05 was considered statistically significant.

## Ethics Statement

The study design and protocol were approved by the Ethics Review Committee of Nippon Medical School (approval number 29-06-767) and written informed consent was obtained from all patients.

## Declaration of Competing Interest

The authors declare that they have no known competing financial interests or personal relationships which have, or could be perceived to have, influenced the work reported in this article.
